# Spectroscopic Analysis on Different Stacking Configurations of Multilayered MoSe_2_

**DOI:** 10.3390/ma17163998

**Published:** 2024-08-11

**Authors:** Xiang Hu, Yong Wang, Jiaren Yuan, Xiaxia Liao, Yangbo Zhou

**Affiliations:** 1 School of Physics and Materials Science, Nanchang University, Nanchang 330031, Chinajryuan@ncu.edu.cn (J.Y.); yangbozhou@ncu.edu.cn (Y.Z.); 2 Jiangxi Key Laboratory for Two-Dimensional Materials, Nanchang University, Nanchang 330031, China

**Keywords:** MoSe_2_, stacking configuration, low-frequency Raman spectroscopy mapping, second harmonic generation, salt-assisted chemical vapor deposition

## Abstract

Transition metal dichalcogenides (TMDs) are drawing significant attention due to their intriguing photoelectric properties, and these interesting properties are closely related to the number of layers. Obtaining layer-controlled and high-quality TMD is still a challenge. In this context, we use the salt-assisted chemical vapor deposition to grow multilayered MoSe_2_ flake and characterize it by Raman spectroscopy, second harmonic generation, and photon luminescence. Spectroscopic analysis is an effective way to characterize the stacking order and optoelectronic properties of two-dimensional materials. Notably, the corresponding mapping reflects the film quality and homogeneity. We found that the grown continuous monolayer, bilayer, and trilayer of MoSe_2_ sheets with different stacking orders exhibit distinctive features. For bilayer MoSe_2_, the most stable stacking configurations are the AA’ and AB order. And the uniformity of the spectroscopy maps demonstrates the high quality of the stacked MoSe_2_ sheets.

## 1. Introduction

Molybdenum Diselenide (MoSe_2_) is a member of the transition metal dichalcogenides (TMD). Compared with the more widely studied MoS_2_, MoSe_2_ has received increasing attentions into the preparations and applications due to a number of highly interesting physical properties, such as the narrow bandgap, unique optoelectronics, etc. [1,2]. And the various stacking orders further enrich the properties of multilayered MX_2_ (M = Mo, W, and X = S, Se) [3,4]. Bulk like 2H symmetry and 3R stacking are confirmed to the two stable configurations of bilayer MX_2_ [5,6,7,8]. In these two configurations, the corresponding electronic, optical, and phonon scattering are significantly modified [4,9]. In the low-frequency Raman spectroscopy, one shear and two breathing vibration modes are detected on 2H and 3R MoSe_2_, and these vibration modes are highly sensitive to the layer numbers and the symmetry [6,10,11,12,13,14]. From bilayer to trilayer MoSe_2_, the characteristic shear mode of 19.2 cm^−1^ disappears and two new modes around 13 cm^−1^ and 24 cm^−1^ appear, which is attributed to different symmetry as well as Raman tensors. By changing the twisted angle from 60° to 59.6° of bilayer MoSe_2_, the breathing B1 mode is observed; when further twisting the angle to 55.3°, the intensity of the prominent shear mode diminishes to zero instead of a strong B2 mode [13]. The symmetry of different stacking configurations can also be demonstrated by the Second harmonic generation (SHG). SHG is a powerful method to characterize the centrosymmetry and inversion symmetry. When the symmetry is broken, a strong SHG signal will be acquired. 2H-stacked MoSe_2_ is with inversion symmetry without SHG response, but 3R-stacked MoSe_2_ is non-centrosymmetric with strong SHG feedback [5,15,16].

In this work, salt-assisted chemical vapor deposition (CVD) is applied to grow the multilayered MoSe_2_. Through controlling the preparation conditions, the thickness (layers) and the stacking order of MoSe_2_ are deliberately tuned. We have successfully grown several multilayered MoSe_2_ with completely opposite stacking patterns. A series of characterizations includes optical microscopy, low-frequency Raman spectroscopy, SHG, and photoluminescence (PL); in addition, corresponding mappings are performed to investigate their stacking order and properties. Density function theory is carried out to verify the stability of the stack order. Low-frequency Raman spectroscopy is a fingerprint of MX_2_ stacking order, and the SHG mapping reflects the uniformity of the sheets.

## 2. Materials and Methods

### 2.1. Salt-Assisted CVD Synthesis of MoSe_2_

**Figure 1:**
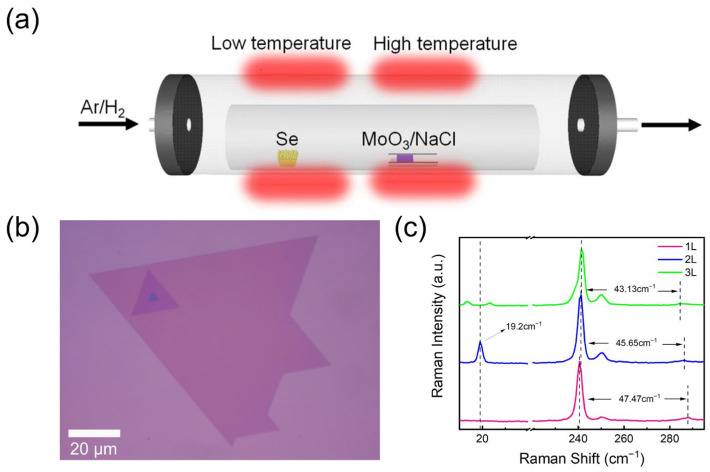
(**a**) The schematic diagram of the salt-assisted CVD setup for MoSe_2_ growth. (**b**) The optical microscopy image of monolayer, bilayer, and trilayer MoSe_2_. (**c**) The corresponding Raman characterization of monolayer (1L), bilayer (2L), and trilayer (3L) MoSe_2_ in (**b**).

The stacked MoSe_2_ sheets were grown by salt-assisted CVD in a two-zone furnace with a 2-inch diameter horizontal quartz tube (OTF-1200X-III-C, Hefei kejing materials technology Co., Ltd., Hefei, China) (Figure 1a). Before putting the 285 nm SiO_2_/Si substrate into the tube, it was cleaned with deionized water, acetone, and isopropyl alcohol consecutively and then dried by N_2_. Two corundum boats filled with excess Se powder (300 mg) and MoO_3_/NaCl mixture (5 mg, with a ratio of 6:1) were placed following the gas stream at the low-temperature and high-temperature zones, respectively. The SiO_2_/Si substrate was directly put above the boat of the MoO_3_/NaCl mixture upside down. During the growth process, a mixed Ar/H_2_ carrier with 150 sccm was introduced, and 5% of the H_2_ was supposed to create a reducing atmosphere to promote the reaction. The pressure was 0.9 atmospheres. In the high-temperature zone with the MoO_3_/NaCl mixture, it was heated from 25 °C to 600 °C with a heating rate of 30 °C/min for 2 min, and then heated to 760 °C at 20 °C/min for 10 min. For the low-temperature zone, it was heated from 25 °C to 350 °C with 10 °C/min and held over 10 min for a sufficient reaction. After the reaction, both temperature zones were naturally cooled down.

### 2.2. Characterizations of Different Stacked MoSe_2_ Sheets

The geometry of different stacked MoSe_2_ sheets was identified by an optical microscope (Nikon Ci-Pol, Minato City, Japan). All optical images were captured from the Raman instrument retention. The structure and optoelectronic properties of different stacked MoSe_2_ were characterized by Raman spectroscopy and photoluminescence spectroscopy. The measuring instrument is alpha 300 Raman produced by WITEC (Ulm, Germany). For all Raman and PL measurements, a microscope lens of 100× and a laser wavelength of 532 nm were used. In order to prevent thermal effects, the laser power of 1 mW with a spot diameter of 1 μm was chosen to avoid damage to the sample. The grating of 1800 g/mm BLZ = 500 nm was selected for the Raman measurements, and 150 g/mm BLZ = 500 nm was grated for the PL measurements. The SHG measurement was performed with a laser wavelength of 1064 nm and the grating of 150 g/mm BLZ = 500 nm.

### 2.3. DFT Calculation of Bilayer MoSe_2_

We performed first-principles calculations with the software package Vienna Ab initio Simulation Package (VASP4.6) [17] based on density functional theory. The Perdew–Burke–Ernzerhof (PBE) function was selected as the exchange–correlation function [18]. To avoid mirror interaction, a 20 Å vacuum layer was adopted. The convergence thresholds for energy and force were set at less than 10^−5^ eV and 0.01 eV/Å, respectively. To account for the vdW force between layers of the bilayer structure, we performed the vdW calibration of DFT-D_3_ [19]. The Brillouin zone (BZ) integration was sampled using a 19 × 19 × 1 k-point grid.

## 3. Results and Discussion

Figure 1a is the schematic diagram of the salt-assisted CVD setup to grow the MoSe_2_ sheets with different stacking orders. The details of the growth procedure are described in the Section 2. As already claimed in the previous studies [20,21], adding salts (such as NaCl, KCl, etc.) as promoters in the evaporated oxide powder can effectively reduce the melting point of the reactants, so as to promote the formation of intermediates and increase the reaction speed. Here, NaCl is mixed into the MoO_3_ precursor. The optical microscopy image of the product is shown in Figure 1b. Few-layer MoSe_2_ sheets are formed by stacking different orientations of typical triangular shapes. The obvious color contrast clearly distinguishes the monolayer-, bilayer-, and trilayer-stacked MoSe_2_, because color variation is a common way to identify the thickness/layers of two-dimensional materials [22]. The deepening color is supposed to concomitantly increase the layer number of MoSe_2_. 

We also perform Raman spectroscopy to characterize the structural properties of the stacked MoSe_2_ sheet. Raman spectroscopy has proven to be a powerful tool for studying the optical response of two-dimensional van der Waals materials as well as the differentiation of layer numbers [23]. As shown in Figure 1c, at the high-frequency mode, MoSe_2_ mainly has two characteristic Raman modes, out of plane A_1g_ (240.5 cm^−1^) and in-plane $E_{2g}^{1}$ (285.5 cm^−1^). The intensity of these two mode peaks is independent of the MoSe_2_ thickness. However, the frequency difference Δ between A_1g_ and $E_{2g}^{1}$ mode decreases gradually from 47.5 cm^−1^ of monolayer to 45.6 cm^−1^ of bilayer and 43.1 cm^−1^ of trilayer MoSe_2_. This trend is consistent with the previous reports and is ascribed to the strong interface coupling [5]. So, the frequency difference is commonly used to define the layer number of TMDs. While at the low-frequency mode, significant changes are observed. For monolayer MoSe_2_, no obvious peak is detected at the low-frequency region. However, a strong peak at 19.2 cm^−1^ appears on the bilayer MoSe_2_, and this peak further splits into two lessened peaks (at 13.4 cm^−1^ and 23.2 cm^−1^) on the trilayer MoSe_2_. This indicates that low-frequency Raman spectroscopy is highly sensitive to the sheet layers. Puretzky and Lu et al. attribute the 19.2 cm^−1^ peak to the shear mode of the bilayer MoSe_2_ and explain the split of the shear mode in trilayer MoSe_2_ to the interlayer bond polarizability mode [6,11,24]. This phenomenon is also observed in the other TMDs, such as MoS_2_ and WSe_2_, and is strongly dependent on the stacking orders as well [24,25]. We will discuss the stacking configuration below. The intensity and symmetry of the Raman peaks in high/low frequency shows that our MoSe_2_ sheet is relatively uniform.

**Figure 2:**
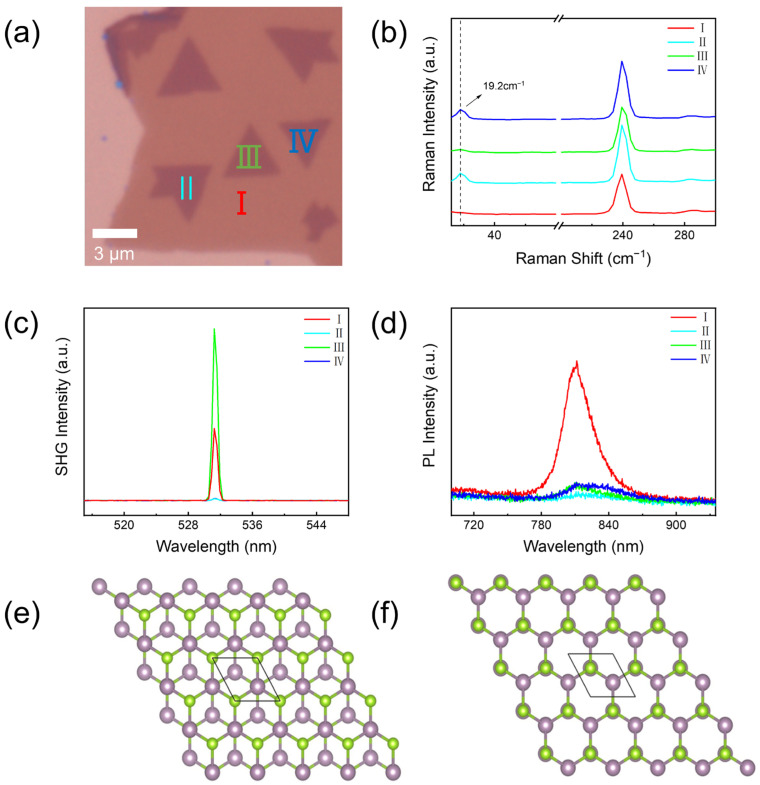
(**a**) Optical microscopy image of bilayer MoSe_2_ with different orientations marked by I, II, III, and IV. The corresponding (**b**) Raman spectroscopy, (**c**) SHG, and (**d**) PL spectra of the four regions. Crystal structure of (**e**) AA’ and (**f**) AB stack configuration of bilayer MoSe_2_.

To obtain detailed insights into the stacking order of the MoSe_2_ sheets, we studied several bilayer MoSe_2_ with completely opposite orientations on the continuous film, as shown in Figure 2a. The 1L of the continuous film is marked by I, and three different orientations of the second triangular MoSe_2_ layer are marked by II, III, and IV. Region II and IV have the same orientation and are antiparallel to region III. Figure 2b is the corresponding Raman spectroscopy. On the high-frequency Raman side, the featured vibration modes (240.5 cm^−1^) with a comparable intensity of MoSe_2_ are observed in these four regions, and it is similar to the one discussed above. On the low-frequency Raman side, monolayer MoSe_2_ in the region I has no signal at all. Bilayer MoSe_2_ in regions II, III, and IV all exhibit a shear mode of 19.2 cm^−1^, but the intensities of regions II and IV are stronger than region III. The low-frequency mode is strongly dependent on the stack order and layer number of TMDs and is significantly influenced by interface coupling, symmetry, and so on [6,11,24]. Therefore, we further conducted the nonlinear optics SHG measurements to investigate the stack order of the bilayer, as shown in Figure 2c. SHG signals are highly affected by the centrosymmetry and inversion symmetry of materials [26]. Monolayer MoSe_2_ belongs to the non-centrosymmetric $D_{3h}^{1}$ group, so region I presents a strong SHG signal [5]. In region III, the SHG signal is strengthened. We speculate the second layer of MoSe_2_ possesses the same orientation as the first layer, so the accumulation of the non-centrosymmetry enhances the SHG intensity. On the contrary, the SHG signal in regions II and IV is highly suppressed. The stack order of these bilayers is supposed to be antiparallel and inversion symmetry. This phenomenon is also reported in the literature. The bilayer without twist (θ = 0°) exhibits a strong SHG signal, while the SHG disappears both in twisted (θ = 60°) bilayer MoSe_2_ and MoS_2_ [5,26,27,28]. We propose the bilayer MoSe_2_ in region III and regions II, IV have a completely different stacking pattern with the twist angle of θ = 0° and θ = 60°, respectively. However, the twist angle has minor effects on the PL spectroscopy of this bilayer MoSe_2_, which displays a similarly weaker intensity compared to the monolayer, as shown in Figure 2d. The monolayer in region I has a main peak at 815 nm converting to a band gap of 1.52 eV. The PL intensity of stacked bilayer MoSe_2_ in three different regions is significantly suppressed due to the indirect-to-direct bandgap transition [5]. DFT calculations are carried out to further confirm the stability of different stacking orders of bilayer MoSe_2_. According to the previous studies [4,8], five typical stacking configurations (AA, AA’, A’B, AB’, and AB) are calculated. We found that the AA’ configuration has the lowest total energy and AB configuration with 2.9 meV/formula unit higher. This result agrees well with ref. [8]. The crystal structure of AA’ and AB configurations are displayed in Figure 2e,f. In the AA’ configuration, the Mo (Se) atom is over the Se (Mo) atom with a twisted angle of 60°, while in AB configuration with θ = 0°, Mo over Se and the other Mo and Se are over the center of the hexagons. To sum up, bilayer MoSe_2_ in region III is stacked by AB configuration, while in regions II and IV, they are stacked by AA’ configuration.

**Figure 3:**
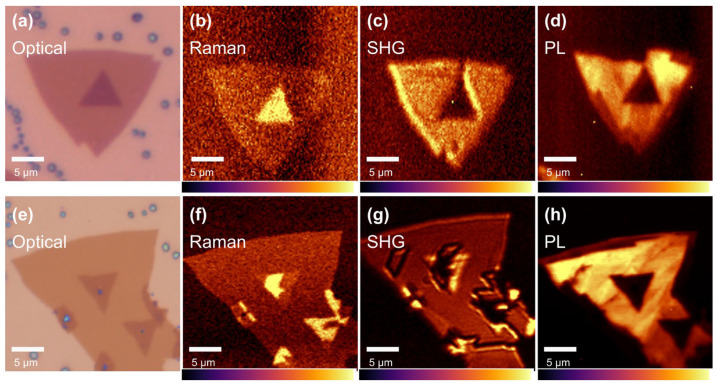
(**a**) Optical microscopy image, (**b**) Raman mapping (19.2 cm^−1^), (**c**) SHG (532 nm) mapping, and (**d**) PL (815 nm) mapping of AA’-stacked bilayer MoSe_2_. (**e**) Optical microscopy image, (**f**) Raman mapping (19.2 cm^−1^), (**g**) SHG (532 nm) mapping, and (**h**) PL (815 nm) mapping of AB-stacked bilayer MoSe_2_.

In order to investigate the natural properties of different stacking MoSe_2_, we perform the Raman, PL, and SHG mappings on a separate AA’ and AB bilayer. Figure 3a,e are the optical microscopy images of AA’- and AB-stacked MoSe_2_. As mentioned above, low-frequency Raman spectroscopy is an efficient method to characterize the stacking order of the TMDs. A strong shear mode of AA’-stacked MoSe_2_ is detected at 19.2 cm^−1^ (Figure 2c of region III), so a Raman mapping with a low wavenumber of 19.2 cm^−1^ is acquired, as shown in Figure 3b of the AA’ stack and Figure 3f of the AB stack. The AA’-stacked MoSe_2_ shows an exceptionally strong and uniform Raman response, while AB-stacked MoSe_2_ displays only a visible contour signal. This evident distinction is ascribed to the change of symmetry and demonstrated by the DFT calculations [6]. However, there is a strong Raman response in the middle of AB-stacked MoSe_2_. This may be explained by the non-uniformity of the MoSe_2_ sheet or defects. Minor deviation of the orientation of bilayer MoSe_2_ leads to great change in the low-frequency vibration mode. Puretzky et al. show that there is a small change of the twisted angle from 60° to 55.3°, the shear mode of bilayer MoSe_2_ has completely disappeared, and the two breathing modes are also highly influenced by the twisted angles [13].

In the SHG mapping, for the AA’ stack, two layers of MoSe_2_ with a twisted angle of 60° belong to an inversion symmetry, so a suppressed SHG signal is observed in Figure 3c. For the AB stack, compared to the monolayer MoSe_2_, a stronger SHG signal is captured due to the enhanced non-centrosymmetry. This discovery of non-uniformity is consistent with the low-frequency Raman mapping of the AB stack. In the PL mapping, as shown in Figure 3d–h, both the AA’ and AB stacks exhibit weaker and even PL properties than the monolayer ones, without the influence of a grain boundary or twisted angle. From these mapping results, the stacking order, uniformity, and quality of the MoSe_2_ layer are revealed.

**Figure 4:**
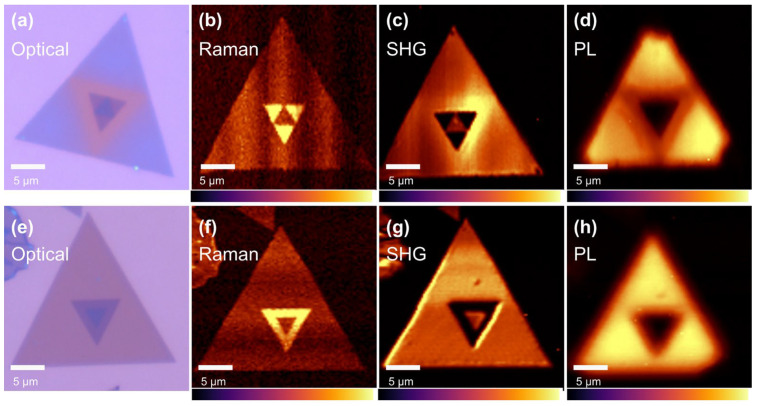
(**a**) Optical microscopy image, (**b**) Raman mapping (19.2 cm^−1^), (**c**) SHG (532 nm) mapping, and (**d**) PL (815 nm) mapping of AA’A-stacked trilayer MoSe_2_. (**e**) Optical microscopy image, (**f**) Raman mapping (19.2 cm^−1^), (**g**) SHG (532 nm) mapping, and (**h**) PL (815 nm) mapping of AA’B-stacked trilayer MoSe_2_.

By further optimizing the growth parameters, we also successfully obtain the perfect trilayer MoSe_2_ with AA’A stack and AA’B stack, respectively, as shown in Figure 4a–e. This complies with the fact that AA’ stack is the most stable configuration. The corresponding Raman mapping is shown in Figure 4b,f. it can be seen that the second layer has strong vibration at 19.2 cm^−1^ in both the AA’A and AA’B stacks, while the intensity is diminished on the third layer. This might be due to the split of the vibration mode into 13.4 cm^−1^ and 23.2 cm^−1^ of the trilayer MoSe_2_, as we mentioned in Figure 1c. Two vibration modes of 13.7 cm^−1^ and 23.1 cm^−1^ are reported in trilayer MoSe_2_ [6,11]. Figure 4c,g shows the SHG mappings. As discussed above, the AA’ stack possesses a suppressed SHG due to the forming of inversion symmetry. But a similar result is not expected in the AA’A stack. The strong SHG of the AA’A stack might have originated from the interaction between the first and third layers. Shinde et al. present a similar result in MoS_2_ [27]. For the PL mapping shown in Figure 4d,h, the intensity is highly affected by the thickness due to the transition of the direct–indirect band gap. In the Raman and SHG mappings, both the AA’A and AA’B stacks appear with shape edges; this indicates a uniform high quality of our trilayer MoSe_2_. But in the AA’B stack, the PL vanishes at three angles. This may need further investigation.

## 4. Conclusions

In conclusion, we have successfully grown the multilayered MoSe_2_ sheets with different stacking orders by salt-assisted CVD. After a series of spectroscopic characterizations and DFT calculations, we have discovered that the AA’ (θ = 60°) and AB (θ = 0°) stacks are stable configurations of bilayer MoSe_2_. These two stack configurations exhibit quite different low-frequency Raman modes and SHG signals due to the change in symmetry. Compared to the bilayer MoSe_2_, the featured Raman mode at 19.2 cm^−1^ splits into two peaks at 13.4 cm^−1^ and 23.2 cm^−1^ of trilayer MoSe_2_ and can be well explained by the interlayer bond polarizability model. So, the low-frequency Raman and SHG are efficient ways to differentiate the stack order and the layer number of MoSe_2_. Moreover, the low-frequency Raman, SHG, and PL mappings obviously reflect the homogeneity and distribution of the bilayer and trilayer MoSe_2_, which provide references to the future optimization of CVD growth.

## Data Availability

The original contributions presented in the study are included in the article, further inquiries can be directed to the corresponding author.
